# Towards routine accurate electron-density studies of biological macromolecules

**DOI:** 10.1107/S2059798326007448

**Published:** 2026-08-12

**Authors:** Elham Paknia, Claus Flensburg, Michal Leszek Chodkiewicz, Rasmus H. Fogh, Peter Keller, Clemens Vonrhein, Clemens Schulze-Briese, Paulina Maria Dominiak, Gleb Bourenkov, Gérard Bricogne, Ashwin Chari

**Affiliations:** a Department of Structural Dynamics Max Planck Institute for Multidisciplinary Sciences Am Fassberg 11 37077Göttingen Germany; b Global Phasing Limited 9 Journey Campus, Castle Park CambridgeCB3 0AX United Kingdom; c Biological and Chemical Research Centre, Faculty of Chemistry University of Warsaw ul. Zwirki I Wigury 101 02-089Warsaw Poland; d DECTRIS Ltd, Täfernweg 1, 5405Baden-Dättwil, Switzerland; e European Molecular Biology Laboratory Hamburg Unit, Notkestrasse 85 22607Hamburg Germany; f Research Group for Structural Biochemistry and Mechanisms Max Planck Institute for Multidisciplinary Sciences Am Fassberg 11 37077Göttingen Germany; Osaka University, Japan

**Keywords:** sub-Ångström macromolecular crystallography, aspherical atom model, deformation density, quantum crystallography, enzyme mechanism

## Abstract

We report here the structure of *Pyrococcus abyssi* rubredoxin determined at a resolution of 0.43 Å. This, to the best of our knowledge, represents the highest resolution protein structure yet determined. Experimentally, the determination of this structure has been made possible by a series of technical innovations and streamlined procedures that are described herein. The structure model refined with spherical scattering factors, the so-called independent atom model (IAM), reveals many positive difference densities that can be attributed to electrons at the midpoint of chemical bonds. To validate the interpretation that these do indeed represent bonding electrons, we have connected the DiSCaMB transferable aspherical atom model (TAAM) library to *BUSTER*. TAAM refinements resolve all positive density features in midpoints of chemical bonds. Extrapolating the findings and methodology reported here, we anticipate that it should now be possible to routinely acquire accurate X-ray diffraction data for quantum crystallography of biological macromolecules. This is particularly relevant to the study of enzyme mechanisms, which are well documented to entail quantum-mechanical phenomena.

## Abstract 2

Here, we describe the determination of the highest resolution structure in the PDB at 0.43 Å resolution. Electron-density maps reveal extensive deformation electron density that is completely accounted for by performing transferable aspherical atom refinements using the DiSCaMB library in *BUSTER*.

## 1. Introduction

Biological macromolecules perform a myriad of chemical reactions within organisms with extremely high efficiency. They do this in a highly energy-efficient manner, operating at ambient temperatures and pressure and physiological pH while retaining high chemo-, regio- and enantioselectivity, in stark contrast to many established synthetic processes. Most importantly, all of the chemistry performed by biological macromolecules occurs in aqueous solution, unlike most organic synthetic reactions. In order to decipher how biological macromolecules achieve this, an understanding of the chemical principles underlying their architecture and functional parts, akin to that of chemical small molecules, is of great interest.

One means to achieve this is to determine sub-Ångström structures of biological macromolecules and directly visualize (quantum-)chemical details in the observed electron density. Achieving sub-Ångström resolution in X-ray crystal structures is beneficial since it allows structural models to be derived without any prior chemical knowledge or stereochemical restraints during refinement as a consequence of the high observable-to-refined-parameter ratio (Blakeley *et al.*, 2015 ▸; Podjarny *et al.*, 2003 ▸; Dauter *et al.*, 1997 ▸). Specific benefits include the ability to detect and model deviations from peptide planarity, distortions from planarity of aromatic ring systems, subtle alternate-conformation networks, H-atom positions in protein and solvent, and a large proportion of the ordered solvent in fully and partially occupied positions. In addition, sub-Ångström resolution structures hold the promise of observing aspherical electron densities and other quantum-mechanical phenomena directly (Kleemiss *et al.*, 2021 ▸; Zarychta *et al.*, 2007 ▸; Guillot *et al.*, 2008 ▸; Woińska *et al.*, 2016 ▸).

Numerous carefully executed and demanding high-quality, high-resolution crystallographic experiments have been carried out on small molecules in the quest to visualize bonding electron densities and their quantitative analysis (for a review, see Krawczuk & Genoni, 2024 ▸). These efforts laid the foundation for the development of a new branch of crystallography, known today as ‘quantum crystallography’, and stimulated the advancement of methods for interpreting diffraction data. This includes the construction of experimentally parameterized electron-density models (Coppens *et al.*, 1971 ▸; Stewart, 1969 ▸; Hirshfeld, 1971 ▸; Hansen & Coppens, 1978 ▸) and wavefunctions (Jayatilaka, 1998 ▸; Grabowsky *et al.*, 2012 ▸), all of which aim to connect experimental measurements with quantum-mechanical descriptions of the electronic structure. Amongst these, the most widely used is the Hansen–Coppens multipole model (Hansen & Coppens, 1978 ▸), in which the atomic electron density around each atomic centre is described by a finite expansion comprising Slater-type radial functions and spherical harmonics. A drawback of this model is its high number of parameters, requiring the use of very high-resolution data (*d*_min_ < 0.5 Å) in order to have a sufficient number of observations against which to refine these parameters. However, it soon became apparent that multipole model parameters for atoms sharing comparable chemical environments and bonding topologies tend to adopt similar values, suggesting that such parameters could be transferred between chemically analogous atoms (Brock *et al.*, 1991 ▸), yielding improved agreement between the model and the experimental data, and resulting in more reliable atomic displacement parameters compared with refinements using spherical scattering factors. Following the proposition to construct databanks of atom types characterized by their typical electron-density parameters (Pichon-Pesme *et al.*, 1995 ▸), a transferable aspherical atom model (TAAM) approach was built (Bąk *et al.*, 2011 ▸). These databanks may be derived from high-quality experimental charge-density studies [Experimental Library Multipolar Atom Model (ELMAM/ELMAM2) (Pichon-Pesme *et al.*, 1995 ▸; Domagała *et al.*, 2012 ▸)], from theoretical simulations partially constrained by experimental observations [University at Buffalo Pseudoatom Databank (UBDB)/Multipolar Atom Types from Theory and Statistical Clustering Databank (MATTS) (Jha *et al.*, 2022 ▸; Koritsanszky *et al.*, 2002 ▸)] or from fully *ab initio* quantum-mechanical calculations [Invariom/Generalized Invariom Database (GID) (Dittrich *et al.*, 2006 ▸, 2013 ▸)]. Alternatively, a more direct strategy can be applied in which the multipole model and the construction of transferable databanks are bypassed altogether. Instead, the molecular wavefunction of the studied system is computed explicitly and used to generate its electron density, which is then partitioned into atomic contributions using the Hirshfeld scheme (Hirshfeld, 1971 ▸). These atom-centred electron-density fragments can subsequently be employed to calculate aspherical atomic scattering factors directly, without the need for empirical parameter fitting. This approach led to the development of the Hirshfeld atom refinement (HAR) method (Jayatilaka & Dittrich, 2008 ▸), which has since evolved into a family of related techniques (Krawczuk & Genoni, 2024 ▸).

For small molecules, commonly stated resolution requirements for being able to observe deformation density are in the range of 0.5–0.7 Å or better (Afonine *et al.*, 2004 ▸; Jelsch *et al.*, 2000 ▸; Guillot *et al.*, 2008 ▸). Accurate electron-density studies of biological macromolecules have been attempted since the 1990s. These have culminated in several heroic efforts resulting in structures in this resolution range, including crambin (Jelsch *et al.*, 2000 ▸; Schmidt *et al.*, 2011 ▸), rubredoxin (Bönisch *et al.*, 2005 ▸; Chen *et al.*, 2006 ▸), aldose reductase (Howard *et al.*, 2004 ▸; Guillot *et al.*, 2008 ▸), high-potential iron–sulfur protein (HiPIP; Hirano *et al.*, 2016 ▸), cellulase PcCel45A (Nakamura *et al.*, 2015 ▸), lysozyme (Wang *et al.*, 2007 ▸), Z-DNA (Malinska & Dauter, 2016 ▸; Brzezinski *et al.*, 2011 ▸) and type III antifreeze protein (Ko *et al.*, 2003 ▸). Out of a total of 251 217 entries, the PDB (Burley *et al.*, 2019 ▸) as of 9th May 2026 contains only 20 entries (15 protein, four Z-DNA and one RNA) in the resolution range 0.5–0.7 Å suitable for accurate electron-density studies. This indicates the degree of technical difficulty involved in reaching this resolution range for structures of biological macromolecules, which includes obtaining high-quality samples, growing high-quality crystals, collecting high-quality X-ray diffraction data within a strict dose limit, producing high-quality refined models and interpreting these models in terms of deformation density compatible with prior knowledge acquired from small molecules.

Here, we describe technical advances towards routine accurate electron-density studies of biological macromolecules, overcoming the aforementioned technical challenges. This has led to a 0.43 Å resolution structure of rubredoxin, for which the associated maps display ample deformation density. We have further implemented a means to routinely refine high-quality macromolecular X-ray crystallo­graphic structures by a transferable aspherical atom model approach. To this end, we connected the pre-computed aspherical density DiSCaMB library (Chodkiewicz *et al.*, 2018 ▸) to *BUSTER* (Bricogne *et al.*, 2017 ▸). We show that TAAM refinement is indeed able to model difference density features pertaining to deformation density. We further show that the adaptation of today’s technical capabilities in macromolecular crystallography can be leveraged for routine accurate electron-density studies.

## 2. Materials and methods

### 2.1. Rubredoxin purification, crystallization and crystal mounting

The expression and purification of rubredoxin were essentially as described in Bourenkov *et al.* (2026 ▸). In brief, the synthetic open reading frame of rubredoxin (W4L, R5S) from *Pyrococcus abyssi*, preceded by the recognition sequence of TEV protease, was cloned into the T7-promoter-based vector pRSET A (Geneart, Regensburg, Germany). The six-histidine-tagged protein was produced in M9 medium in BL21-AI cells for 3 h at 37°C. The *Escherichia coli* pellet obtained by centrifugation of the culture at 5000*g* for 15 min was resuspended in 10 m*M* Tris–HCl pH 8.0, 10 m*M* imidazole, 200 m*M* NaCl, the cells were lysed by sonication and the lysate was cleared by centrifugation at 100 000*g*. The clear lysate was then loaded onto an Ni^2+^–NTA affinity column (Qiagen), and the column was washed with resuspension buffer and eluted with resuspension buffer containing 500 m*M* imidazole. After hydrolysis of the histidine tag by TEV protease, the protein was again loaded onto an Ni^2+^–NTA affinity column, collected in the unbound fraction and concentrated to less than 5 ml. In the last step, the concentrated protein was loaded onto a HiLoad 26/600 Superdex 75 pg column (Cytiva) equilibrated with 20 m*M* Tris–HCl pH 8.0, 150 m*M* NaCl, 0.1 m*M* EDTA. Finally, pure rubredoxin, as judged by SDS–PAGE and homogeneous elution from gel-permeation chromatography, was concentrated to 36 mg ml^−1^ in 20 m*M* Tris–HCl pH 8.0 and stored at −80°C.

Crystals were grown in 3.6–3.8 *M* sodium malonate pH 6.0 using the sitting-drop vapour-diffusion method at 291 K and had a solvent fraction of 27% and a Matthews coefficient (*V*_M_) of 1.8 Å^3^ Da^−1^. Crystals appeared between one and two weeks after and were ready for harvesting after 14 days. An ortho­rhombic crystal of *P. abyssi* rubredoxin with approximate dimensions of 600 × 500 × 300 µm was mounted in a MiTeGen Dual Thickness MicroLoop (Jena Bioscience, Jena, Germany) and directly cryo-cooled by plunging into liquid nitrogen.

### 2.2. X-ray data collection and processing

**Figure 1:**
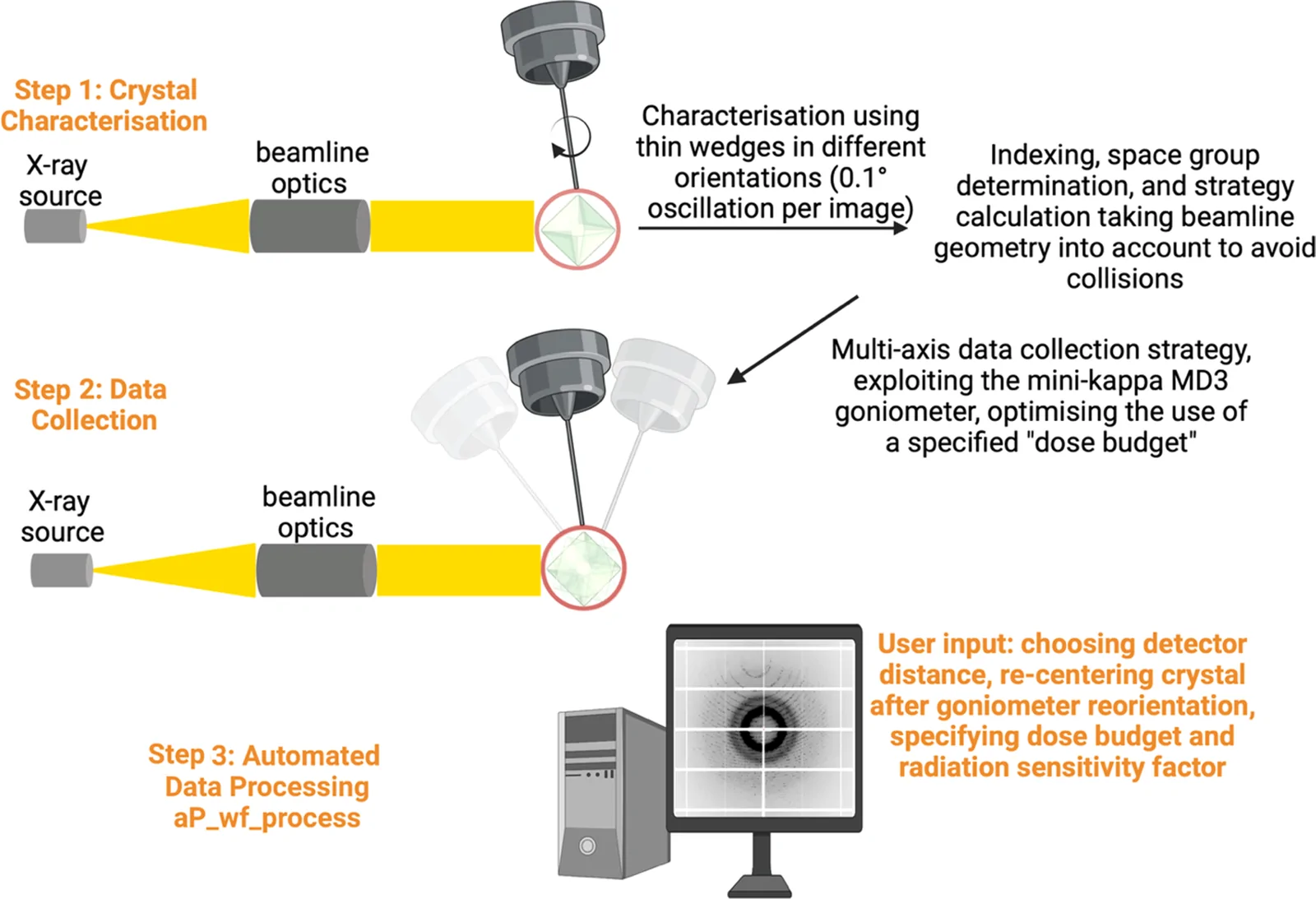
Graphical representation of the *Global Phasing Ltd* (*GPhL*) workflow (Fogh *et al.*, 2026 ▸). The *GPhL* data-collection workflow starts with crystal characterization (step 1). For this, 1.2° thin wedges are collected at 0°, 45°, 90° and 180° with ω-slicing of 0.1° at the PETRA III P14 beamline. These images are indexed using *XDS*, the apparent space group determined and a strategy is calculated to collect data covering the Bragg sphere in reciprocal space in an optimal manner, while avoiding collisions and goniostat shadows. These require the user to specify detector distance, a dose budget and a radiation-sensitivity factor. The data are collected according to a multi-orientation strategy computed by the workflow software, making use of the capabilities of the multi-axis goniostat. Each time the crystal is reoriented by the κ and φ axes (step 2), the user is required to recentre the crystal. The data are then processed in an automated manner using the *aP_wf_process* interface to *autoPROC* (step 3).

Diffraction data were collected at 100 K at an energy of 32.142 keV (*i.e.* a wavelength of 0.3857 Å) with a 601 × 507 µm top-hat beam at full transmission (1.08 × 10^12^ photons s^−1^) on the EMBL Hamburg P14 beamline at the DESY PETRA III storage ring, Hamburg, Germany. The top-hat beam, which was slightly larger than the crystal dimensions, was conditioned by a double-crystal monochromator and by 19 beryllium compound refractive lenses (500 µm apical radius), followed by proximal slits (20 cm from the sample) matching the largest crystal projection. Thus, homogeneous illumination of the crystal was achieved in all orientations during data collection, with a total dose of 500 kGy as estimated by *RADDOSE*-3*D* version 5.0.1057 (Zeldin *et al.*, 2013 ▸; Bury *et al.*, 2018 ▸; Dickerson *et al.*, 2024 ▸). The optimal dose for sub-Ångström resolution data collection was determined earlier (Bourenkov *et al.*, 2026 ▸). The typical beam profile achieved in this setup is shown in Bourenkov *et al.* (2026 ▸). Data collection was designed and executed by the *Global Phasing Ltd* (*GPhL* for short) workflow (Fogh *et al.*, 2026 ▸) as implemented in combination with *MXCuBE* (Oscarsson *et al.*, 2019 ▸). An automatically determined three-sweep strategy was used in which the three goniostat reorientations were chosen on the basis of the crystal symmetry and initial orientation as determined from a characterization pass (see Fig. 1 ▸ for a visual representation). The orientations were selected to fill each other’s cusp regions, to avoid shadowing by the goniostat and to evenly distribute multiplicity and Lorentz-factor enhancement over reciprocal space. Diffraction data collection at these high energies was made possible by an EIGER2 CdTe 16M detector (DECTRIS Ltd, Baden, Switzerland; Donath *et al.*, 2023 ▸) placed at a distance of 139.4 mm from the sample position.

Data processing was carried out in a manner tightly coupled to the designed strategy used for data collection, using *XDS* (Kabsch, 2010 ▸), *AIMLESS* (Evans & Murshudov, 2013 ▸) and *STARANISO* (Tickle *et al.*, 2018 ▸) within the 20250717 version of *autoPROC* (Vonrhein *et al.*, 2011 ▸). Diffraction limits were determined after applying a cutoff of 1.2 to the local average of *I*/σ(*I*), as implemented in *STARANISO*. The use of a crystal fully bathed in a top-hat beam provides the unique advantage that the accumulated dose is spatially uniform throughout the crystal at all times, so that the contributions to the total intensity from all regions of the crystal (i) are affected by the same decay *B* factor of typically 1 Å^2^ per MGy of accumulated dose (Borek *et al.*, 2007 ▸; Kmetko *et al.*, 2006 ▸; Leal *et al.*, 2011 ▸) and (ii) have equal weight as a result of the spatially uniform illumination by the beam. As this dose-dependent decay is concordant with the *AIMLESS* scaling model involving restrained *B* factors for thin wedges of images, the correction of intensity decay through internal scaling can take place under optimal conditions (Bourenkov *et al.*, 2026 ▸).

### 2.3. Structure refinement

The cell constants of the crystal were nearly identical to those of the previously determined structure of the W4L, R5S mutant of *P. abyssi* rubredoxin (PDB entry 1yk4; Bönisch *et al.*, 2005 ▸). Therefore, we proceeded directly with refinement using this model in *BUSTER* (Bricogne *et al.*, 2017 ▸) using the *aB_autorefine* interface. Some alternate conformations were added manually in *Coot* (Emsley & Cowtan, 2004 ▸) and further refinement in *BUSTER*, using individual anisotropic atomic displacement parameters (ADPs) and occupancy refinement, yielded the final model (Table 1 ▸).

In the final stage of maximum-likelihood refinement, positional parameters for all atoms were refined using typical restraints for bonds, angles, torsions, planes and nonbonded contacts (Engh & Huber, 2012 ▸). H atoms had an isotropic *B* factor refined (strongly restrained to the isotropic *B*-factor component of their parent atom), while all other atoms had anisotropic ADPs refined using several sphericity and similarity restraints (1–2, 1–3, 1–4 and nonbonded). Occupancies for atoms in alternate conformations (restrained to sum to 1.0), as well as for those modelled as partially occupied (unrestrained in value), were also refined. Water molecules that only had partial occupancy and were modelled with one alternate location identifier only (either A or B) were not restrained in occupancy. The relative weights of the restraints acting upon those model parameters (*X*, *Y*, *Z*, *B*/ADP and occupancy) were left at the defaults used in *BUSTER* for any refinement. For independent atom model (IAM) refinements, automatic adjustment of the weight of the X-ray term relative to the geometry terms (based – in a resolution-dependent manner – on the r.m.s.d. of observed bond distances relative to the standard Engh & Huber ’99 values; Engh & Huber, 2012 ▸) yielded a value of 42.5. This is approximately four times larger than for a typical, sub-1 Å resolution dataset encountered in the PDB archive, and is indicative of an exceptional level of agreement between very high-resolution data and a refined model with very high-quality geometry, allowing a very high weight to be given to the X-ray data without creating a conflict with geometric restraints. A value of 50.0 would represent negligibly restrained refinement. H atoms were treated differently from other atoms, following the standard *BUSTER* ‘hybrid’ model for H atoms: (i) the (*X*, *Y*, *Z*, *B*_iso_) restraints active on them are significantly stronger and (ii) their bond distances are restrained to the nuclear position, except in the X-ray structure-factor calculation, where they are shifted to the electron-cloud position. We have built water molecules up to a level of 1 r.m.s. (1 e Å^−3^, 2*mF*_o_ − *DF*_c_). This is warranted because the noise level of the electron density is spectacularly low. The validity of this water molecule-modelling approach was verified by omit maps and the reappearance of signals above a threshold of 3 r.m.s. (0.7 e Å^−3^) in *mF*_o_ − *DF*_c_ maps. It is noteworthy that positive electron density continued to reappear when the respective partially occupied water molecules were not built. No H atoms were attached to water molecules in this study.

Aspherical scattering factors were computed with the DiSCaMB library (Densities in Structural Chemistry and Molecular Biology; Chodkiewicz *et al.*, 2018 ▸), with multipole parameters being drawn from the MATTS databank (Jha *et al.*, 2022 ▸). Parameters were assigned automatically by atom type according to the chemical environment of each atom in the model. Atoms that were not automatically assigned a type, which included all atoms of the Fe–S_4_ cluster, sodium ions and all water molecules, were described with usual IAM scattering factors. Every other atom, including atoms in disorder, was assigned a TAAM scattering factor. The DiSCaMB library was compiled and linked as an external structure-factor engine within the *BUSTER* refinement package, release 20260424 (Bricogne *et al.*, 2017 ▸), replacing the default structure-factor engine while retaining all other *BUSTER* functionality. Anisotropic displacement parameters (ADPs) were refined for all non-H atoms, while H atoms were refined with isotropic displacement parameters that were restrained to that of their parent atom. In TAAM refinements, the weight of the X-ray term was higher than in IAM refinement, with a value of 47.9, which is close to the X-ray weight of 50 in negligibly restrained refinements. When using standard spherical scattering factors, refinement was performed using a reciprocal-space Fourier summation technique (Bricogne, 2010 ▸) newly implemented in the *BUSTER* 20260424 release. In IAM refinements, Fe^3+^, Na^+^ and S^−^ scattering factors were used. Both IAM and TAAM refinements in *BUSTER* used the occupancy-aware masking for bulk-solvent correction ‘BUSTER-GEMMI’, first implemented in the 20250717 release of *BUSTER*, to handle disordered parts of the unit cell. Water molecules were not assigned aspherical scattering factors in TAAM refinement, as H atoms were not attached in this study.

## 3. Results and discussion

### 3.1. Sample requirements for accurate electron-density studies

The technical challenges for accurate electron-density studies of biological macromolecules outlined in Section 1 prompted us to revisit this topic, especially since a multitude of technical improvements have appeared in recent years. With regard to sample preparation, advances in molecular biology and recombinant expression technologies in comparison to the mid-1990s are indisputable (Derewenda, 2004 ▸; Gorda *et al.*, 2021 ▸; Schütz *et al.*, 2023 ▸; Martínez-Solís *et al.*, 2019 ▸). The same applies to protein-purification capabilities and techniques. Protein quality can routinely be quantified and optimized using biophysical techniques, which improves protein stability and solubility (Atsavapranee *et al.*, 2021 ▸; Chari *et al.*, 2015 ▸; Sumida *et al.*, 2024 ▸; Magliery, 2015 ▸; Deller *et al.*, 2016 ▸; Gao *et al.*, 2020 ▸; Wu *et al.*, 2025 ▸; Asor *et al.*, 2025 ▸). Biophysical techniques used in this regard include differential scanning calorimetry (DSC), differential scanning fluorimetry (DSF)/Proteoplex, mass photometry (MP), circular-dichroism (CD) spectroscopy, UV absorbance and intrinsic fluorescence, to name a few. Taken together, these have made the production of homogeneous samples much more routine. While sample quality is undeniably the most important prerequisite towards the undertaking of accurate electron-density studies, the topic is so vast that it would exceed the scope of this article. Instead, we refer the interested reader to the large body of literature related to this topic (Vijayachandran *et al.*, 2011 ▸; Mesa *et al.*, 2013 ▸; Maeda & Schertler, 2013 ▸; Byrne, 2015 ▸; Barford *et al.*, 2013 ▸; Almo *et al.*, 2013 ▸; Young *et al.*, 2012 ▸). The second step towards accurate electron-density studies is obtaining high-quality crystals that are sufficiently well ordered to diffract to sub-Ångström resolution. This topic is also widely discussed in the literature and has benefited from automation, from a variety of crystallization screens and from miniaturization to consume less sample (McPherson & Kuznetsov, 2014 ▸; McPherson & Cudney, 2014 ▸; Deller & Rupp, 2014 ▸; García-Ruiz *et al.*, 2016 ▸; D’Arcy *et al.*, 2014 ▸; Luft *et al.*, 2014 ▸; Fazio *et al.*, 2014 ▸; Shaw Stewart & Mueller-Dieckmann, 2014 ▸).

### 3.2. Biological macromolecule crystallization for accurate electron-density studies

However, we would like to point out that accurate electron-density studies are accompanied by very stringent demands on protein crystal quality. Achieving resolutions in the range of 0.5–0.7 Å or better requires average *B* factors of ∼5 Å^2^ and lower (Blakeley *et al.*, 2015 ▸; Podjarny *et al.*, 2003 ▸; Dauter *et al.*, 1997 ▸), equivalent to root-mean-square (r.m.s.) displacements of atomic positions throughout the crystalline lattice of 0.25 Å or less. We and others (unpublished work) have found that routinely achieving sub-Ångström resolution requires the reproducible availability of large crystals that exceed 250 µm in size in all three dimensions. This follows directly from the stringent requirements of performing low-dose experiments. It is nearly impossible to grow crystals with dimensions greater than 250 µm in size from the sub-microlitre droplets that are customary in high-throughput setups, as a consequence of the low mass fraction of protein in such crystallization drops (McPherson & Cudney, 2014 ▸). Rather, it is beneficial to employ crystallization setups in volumes ranging from 2 to 20 µl as customary in 24-well formats. Control over subtle variations of protein to crystallization mother-liquor ratios, which is essential to obtaining reproducible crystals of this size, also becomes simpler in such formats.

### 3.3. Preparation of crystals for data collection for accurate electron-density studies

In line with this, we also find it more beneficial to collect a dataset from a single large crystal rather than to merge data collected from several smaller crystals. We are currently unaware of serial crystallography data extending to 0.5–0.7 Å resolution, and the strict requirements for very high isomorphism (0.25 Å r.m.s. displacements as discussed above) can only be fulfilled by collecting data from a single, well-ordered crystal rather than by merging data from multiple, potentially non-isomorphous, crystals. Relatedly, as data collection at cryogenic temperatures expands the radiation-dose tolerance of crystals by more than two orders of magnitude in comparison to room temperature, it is mandatory to collect data for accurate electron-density studies under cryo-conditions. For more information on how radiation damage affects data collection, the interested reader is referred to the existing literature (Garman & Weik, 2023 ▸; Bourenkov *et al.*, 2026 ▸). The detailed electronic structure that accurate electron-density studies aim to reveal is essentially invariant with temperature (Jarzembska *et al.*, 2017 ▸; Zhurov *et al.*, 2011 ▸). The electron density itself, however, will become more diffuse at elevated temperatures (Garman & Weik, 2023 ▸). This dependence on cryocooling increases the demands imposed on post-crystallization treatments, including cryoprotection strategies (Garman & Schneider, 1997 ▸). It is imperative to find suitable stabilization conditions for crystals before unsealing drops, as rapid changes in relative humidity can cause distortion of the lattice-packing interactions, leading to crystal imperfections. Likewise, as cryoprotectants induce dehydration and crystal transformations (Bowler *et al.*, 2017 ▸), it is important, and even crucial, to slowly transfer drops from stabilization to cryoprotection conditions, procedures that can take days in extreme cases. In this context, it is imperative to mention that low mosaicity is a prerequisite to achieving sub-Ångström resolution diffraction.

### 3.4. Diffraction data collection for accurate electron-density studies

#### 3.4.1. Advantages of top-hat beams over Gaussian profiles and detectors

As diffraction spots from these large crystals will necessarily span large intensity ranges, and the highest-resolution reflections will be the weakest, it is crucially important to minimize the non-Bragg scattering during data collection. This holds true for any macromolecular crystallographic experiment at low dose and is due to the fact that the Bragg scattering at the diffraction limit is small, giving rise to only a few counts per pixel. Therefore, minimizing the background scattering is essential. We have repeatedly found that maintaining crystals in a hydrated state, while aiming at reducing the amount of surrounding liquid, is beneficial. In our hands, microfabricated Kapton mounts (MiTeGen, Molecular Dimensions) have proved advantageous in achieving this reproducibly. Crystals mounted in this manner aid multi-orientation data-collection strategies by removing the background scatter in all directions and facilitating the recentring of crystals after goniostat reorientation. The benefit of large, well-centred crystals with a minimal layer of mother liquor is to use the entire volume for diffraction, for which two scenarios can be envisaged: (i) scanning the crystal with a small, focused beam or (ii) exposing the crystal to a beam with a homogenous top-hat profile that matches the crystal dimensions. While the first scenario appears more in line with the design philosophy of most third- and fourth-generation synchrotron beamlines, the second strategy is the only method capable of delivering high-quality data, by virtue of considerations described elsewhere as the ‘Humpty-Dumpty problem’ (Bricogne, 2020 ▸). At the PETRA III P14 beamline, methods for generating large top-hat beams using coherent X-ray optics (compound refractive lenses; CRLs; Snigirev *et al.*, 1996 ▸) were established previously (Schrader *et al.*, 2016 ▸; Singh *et al.*, 2020 ▸) and more recently adapted to high X-ray energies of 20–32 keV. The use of this energy range is imperative for data collection at 0.5–0.7 Å resolution as a consequence of Bragg’s law. While this was already achievable with second-generation synchrotrons and has been extensively pursued (Bönisch *et al.*, 2005 ▸; Schmidt *et al.*, 2011 ▸), the major impediment at the time was that no suitable detectors for X-rays at these energies were available. This has recently changed with the introduction of high element number (high-*Z*) detectors (Pennicard *et al.*, 2017 ▸) that have increased the detective quantum efficiency (DQE) at higher X-ray energies. At the PETRA III P14 beamline, the EIGER2 CdTe 16M detector (DECTRIS Ltd, Baden, Switzerland; Donath *et al.*, 2023 ▸) has been in use since 2021. Its high dynamic range and DQE at high X-ray energies offers the extra benefit that it allows the collection of the entire resolution range in a single pass even when aiming at very high resolution (Donath *et al.*, 2023 ▸; Pennicard *et al.*, 2017 ▸).

#### 3.4.2. Strategies for high-quality data collection

For high-resolution data collection, two additional points need to be taken into account: first, the use of high-multiplicity data collection, made possible by fast-readout detectors and continuous rotation, is imperative in order to achieve high precision (Mueller *et al.*, 2012 ▸), and second, data-collection strategies should aim at avoiding cusps, which are prevalent in single-orientation datasets and diminish completeness in data collection (Dauter, 1999 ▸; Fogh *et al.*, 2026 ▸). While solutions to both issues were in principle available through multi-axis goniostats for many years, their practical use required manual operation and was challenging, which discouraged users from attempting optimized multi-orientation data-collection strategies. As a result, most datasets were collected in a single orientation and tended to display cusps and low, non-uniformly distributed multiplicity. The goal of automating (and therefore making routine) the use of multi-axis goniostats in the design and execution of optimized strategies was achieved by Global Phasing (Fogh *et al.*, 2026 ▸). Their workflow has been connected to the *MXCuBE* beamline-control software of the PETRA III P14 beamline, enabling the on-the-fly design and execution of data-collection experiments that routinely employ multiple orientations, thus avoiding cusps and allowing high-multiplicity data collection (see Fig. 1 ▸ for a graphical representation). These workflows also provide a full instrument description, which is particularly important in multi-orientation datasets because of potential goniostat shadows at high χ angles when short detector distances are used (see Section 4.2 and Figs. 6 and 7 in Fogh *et al.*, 2026 ▸). The instrument description then allows the generation of per-image lists of shadowed reflections that are passed through a workflow-specific interface to the fully automated *autoPROC* pipeline, where they are removed from the scaling step, ensuring consistent, shadow-free multi-sweep data processing.

### 3.5. Rubredoxin at 0.43 Å resolution

#### 3.5.1. Independent atom model (IAM) refinement

**Figure 2:**
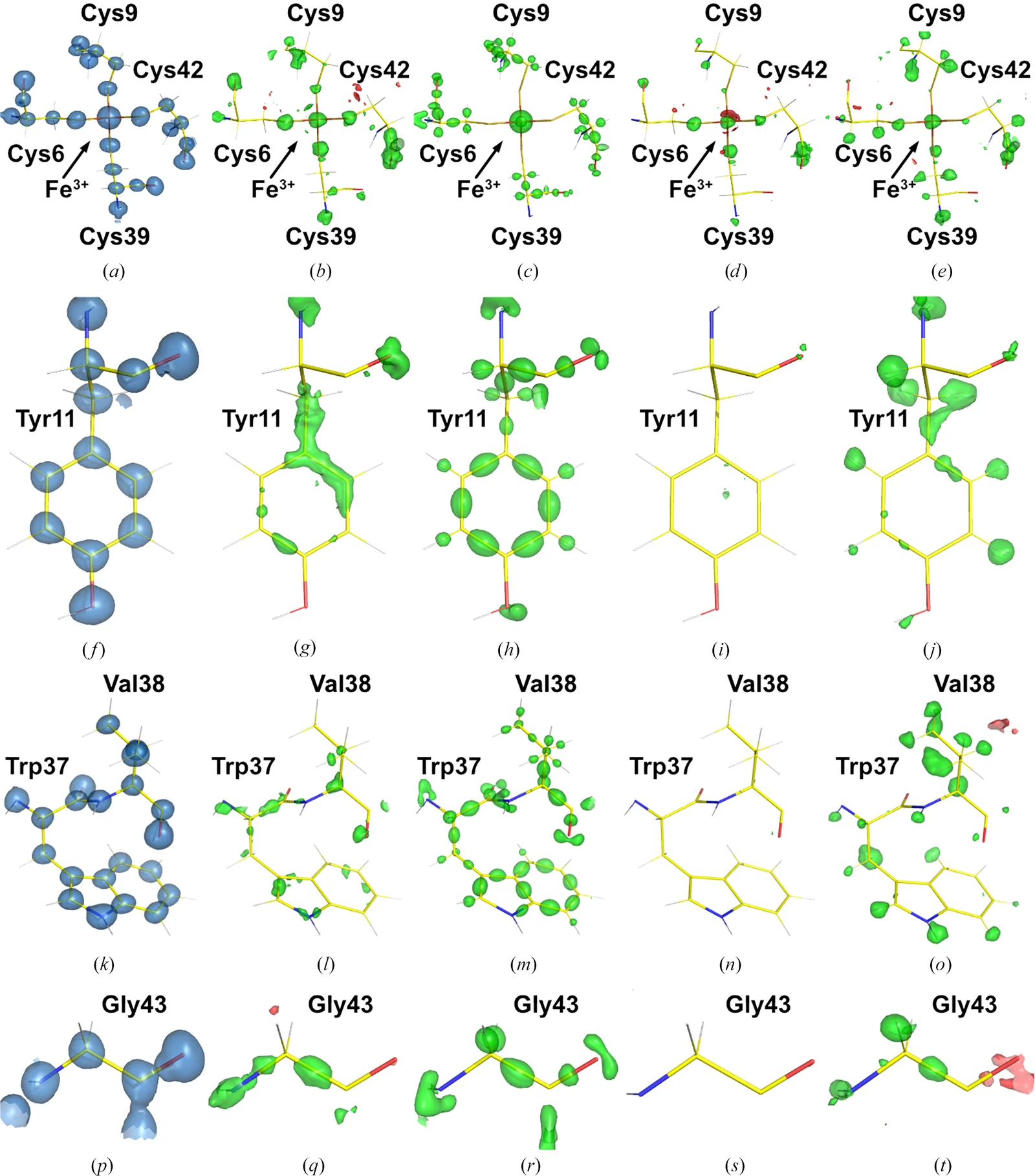
Genuine deformation density in the rubredoxin structure at 0.43 Å resolution. Depicted are 2*mF*_o_ − *DF*_c_ maps contoured at 2.0 e Å^−3^ in (*a*), (*f*), (*k*) and (*p*), *mF*_o_ − *DF*_c,IAM_ maps contoured at ±0.55 e Å^−3^ in (*b*), (*g*), (*l*) and (*q*), deformation density (*F*_c,TAAM_ − *F*_c,IAM_) maps contoured at ±0.25 e Å^−3^ in (*c*), (*h*), (*m*) and (*r*), and *mF*_o_ − *DF*_c,TAAM_ maps contoured at ±0.55 e Å^−3^ in (*d*), (*i*), (*n*) and (*s*), as well as *mF*_o_ − *DF*_c,IAM_ (hydrogen-omit) maps contoured at ±0.8 e Å^−3^ in (*e*), (*j*), (*o*) and (*t*). In (*a*)–(*e*) the Fe–S_4_ site is shown, in (*f*)–(*j*) Tyr11, in (*k*)–(*o*) the Trp37-Val38 dipeptide and finally in (*p*)–(*t*) Gly43. Note that the majority of positive difference density visible in *mF*_o_ − *DF*_c,IAM_ maps resembles features in the deformation map, and these features are not present in the *mF*_o_ − *DF*_c,TAAM_ maps, providing evidence that *mF*_o_ − *DF*_c,IAM_ map density features indeed represent deformation density. The Fe–S_4_ site was refined with IAM scattering factors, as no aspherical descriptions exist in the MATTS databank. All maps were computed by rejecting 383 reflections, which corresponds to a log-likelihood level of −4.

**Figure 3:**
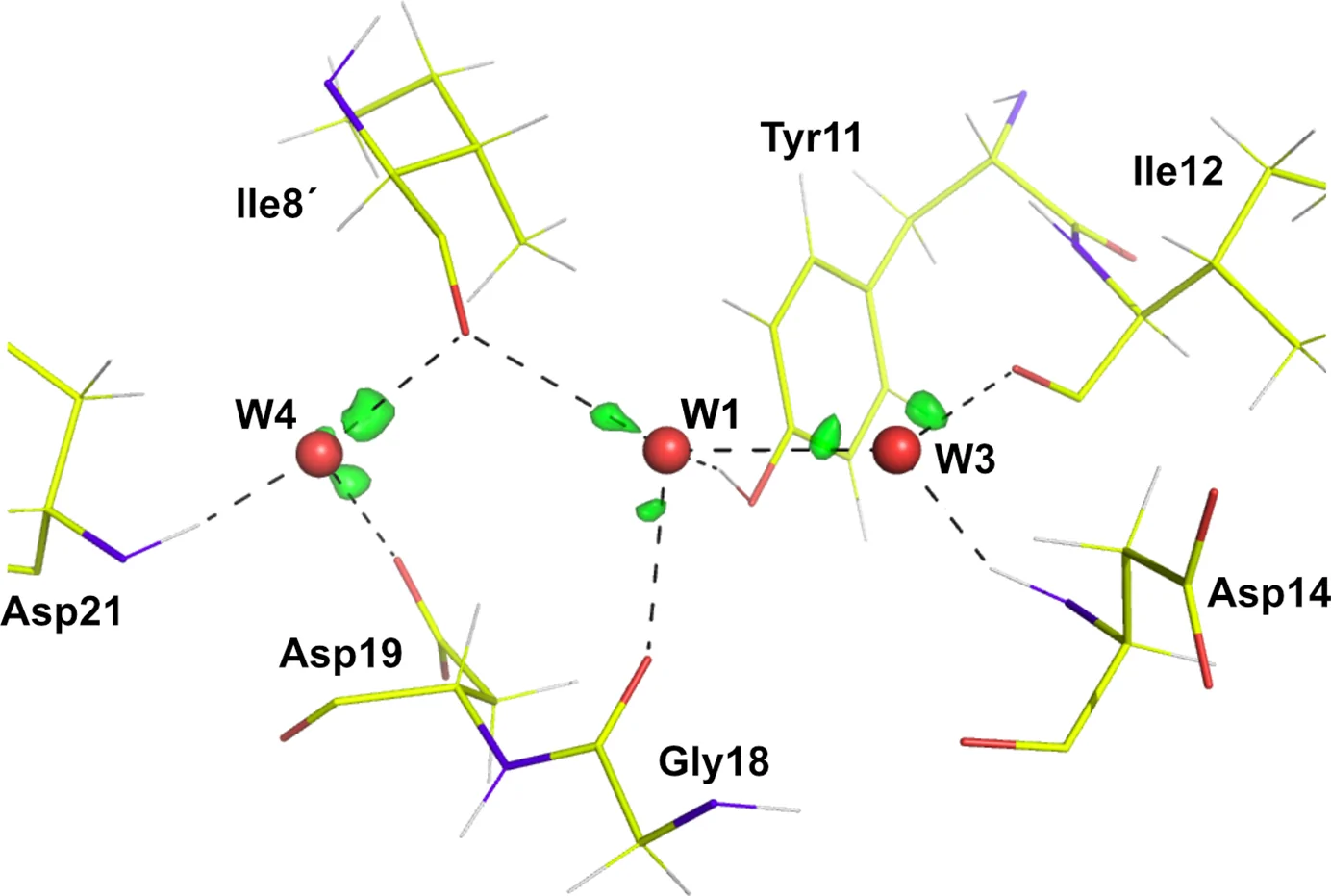
Visualization of water H atoms to complete hydrogen-bonding networks. Shown are water molecules W1, W3 and W4 along with their surrounding *mF*_o_ − *DF*_c,TAAM_ map contoured at ±0.8 e Å^−3^. The immediate hydrogen-bonding environment of these three water molecules is also illustrated and includes Tyr11, Ile12, Asp14, Gly18, Asp19, Asp21 and Ile8 from an adjacent symmetry mate. Note that none of the water molecules were hydrogenated in refinement.

We used this setup to collect rubredoxin data in the resolution range 20.921–0.433 Å with a total dose of 500 kGy. The results of data processing and refinement are given in Table 1 ▸. We collected 6 545 565 total and 245 905 unique reflections, resulting in a multiplicity of 26.6. Standard procedures for computing the Wilson *B* factor report a value of 1.94 Å^2^ for this dataset (Agirre *et al.*, 2023 ▸), whereas the *STARANISOB* eigenvalues correspond to 4.9 Å^2^ along the a*, 4.5 Å^2^ along the b* and 4.5 Å^2^ along the c* directions (Tickle *et al.*, 2018 ▸). It appears that there is a need to re-evaluate the Wilson plot at sub-Ångström resolution. The data are precise and significant to the resolution limit, with a CC_1/2_ of 0.621 in the outer shell and an *I*/σ(*I*) of 1.7 (Table 1 ▸). The model was refined with ADPs and independent atom model (IAM) scattering factors to an *R* and *R*_free_ of 6.82% and 7.25%, respectively. The final model contains the entire chain of *P. abyssi* rubredoxin (W4L, R5S) preceded by three amino acids remaining from tag cleavage, one Fe^3+^ ion, three Na^+^ ions, two of which have partial occupancy, and 115 water molecules, with 58 in alternate conformations. The average model *B* factor was 4.8 Å^2^, the average main-chain *B* factor was 2.9 Å^2^, the average side-chain *B* factor was 3.8 Å^2^ and the average water *B* factor was 10.1 Å^2^. All protein H-atom positions were confirmed by hydrogen-omit maps (Figs. 2 ▸*e*, 2 ▸*j*, 2 ▸*o* and 2 ▸*t*), and H atoms on several water molecules were visible as positive difference density in *mF*_o_ − *DF*_c_ maps (Fig. 3 ▸). We have not hydrogenated any water molecules in the model.

The four Fe–S distances of the Fe–S_4_ site of rubredoxin (Figs. 2 ▸*a*–2 ▸*e*) were found to be 2.299 (2) Å (Fe–Cys6 Sγ), 2.270 (2) Å (Fe–Cys9 Sγ), 2.338 (2) Å (Fe–Cys39 Sγ) and 2.277 (2) Å (Fe–Cys42 Sγ) in the model, with an average of 2.296 Å. The standard uncertainty in parentheses was estimated using the diffraction precision index (DPI) method based on *R*_free_ (Cruickshank, 1999 ▸; Blow, 2002 ▸; Gurusaran *et al.*, 2014 ▸). Based on our previous radiation-damage studies on rubredoxin (Bourenkov *et al.*, 2026 ▸), the four Fe–S distances lie precisely between those of the 50 kGy and 1 MGy structures, signifying that Fe^3+^ is partially reduced to Fe^2+^. After an initial ADP and occupancy refinement using the IAM scattering factor model in *BUSTER*, we rejected 383 reflections based on a log-likelihood (LL) cutoff level of −4. The procedures for model description and parametrization at our disposal, and used during refinement, resulted in feedback from the maximum-likelihood (ML) refinement in *BUSTER* about data-model disagreements via per-reflection LL values. Those reflections do not agree with the best model parametrization at our disposal, which could point to future improvements in model parametrization, especially in relation to disordered solvent modelling. Thereby, in the 20.921–6.0 Å resolution shell 32% of reflections were rejected. Otherwise, the remaining LL outliers were uniformly distributed amongst resolution shells. Rejection of these LL outlier reflections markedly increased the contrast of *mF*_o_ − *DF*_c_ difference density maps calculated with the IAM scattering-factor model. This revealed nearly 50% of the electron density on the midpoint of carbon–carbon, carbon–nitrogen and carbon–oxygen bonds, and valence electrons as positive differences above 2.7 r.m.s., which corresponds to 0.55 e Å^−3^ (Figs. 2 ▸*b*, 2 ▸*g*, 2 ▸*l* and 2 ▸*q*). Note that although both positive and negative difference densities are shown, within the entire modelled rubredoxin protein molecule, there was no interpretable negative difference density.

#### 3.5.2. Transferable aspherical atom model (TAAM) refinement

In order to verify that these positive difference densities indeed represented genuine deformation electron density, we performed refinements in *BUSTER* using transferable aspherical atom model (TAAM) scattering factors. To this end, we used the DiSCaMB library to compute aspherical scattering factors, draw multipole parameters from the MATTS databank and assign them automatically by atom type (see Section 2 for details). The model refined with aspherical atoms using TAAM had an *R* and *R*_free_ of 6.51% and 6.93%, respcetively. The final model contains the entire chain of *P. abyssi* rubredoxin (W4L, R5S) preceded by three amino acids remaining from tag cleavage, one Fe^3+^ ion, three Na^+^ ions, two of which have partial occupancy, and 115 water molecules, with 58 in alternate conformations. The average model *B* factor was 4.7 Å^2^, the average main-chain *B* factor was 2.9 Å^2^, the average side-chain *B* factor was 4.2 Å^2^ and the average water *B* factor was 10.5 Å^2^. In all instances, the *mF*_o_ − *DF*_c_ density map did not show any features when contoured at 0.55 e Å^−3^, which corresponds to 2.8 r.m.s. (Figs. 2 ▸*d*, 2 ▸*i*, 2 ▸*n* and 2 ▸*s*). Thus although no impressive drop in *R* factors or significant changes in *B* factors were observed (after IAM refinement), TAAM-based refinement validated the positive differences observed on the midpoint of carbon–carbon, carbon–nitrogen and carbon–oxygen bonds, and valence electrons as deformation density. This is also consistent with the small differences between IAM and TAAM model structure factors (*F*_c_), which have an *R* factor of 2.1% and an average phase shift of 1.1°. Having verified that positive difference densities indeed represented experimental deformation densities, we next computed a deformation electron-density map for the entire rubredoxin model (Figs. 2 ▸*c*, 2 ▸*h*, 2 ▸*m* and 2 ▸*r*). For this purpose, we computed *F*_c,TAAM_ using the TAAM refined model and then calculated *F*_c,IAM_ from the same model. The map computed from the *F*_c,TAAM_ − *F*_c,IAM_ vector-difference coefficients then represents all valence-induced electron-density deformations within the structure. This demonstrates that our experimentally observed positive difference densities, although noisy, do represent genuine features elicited by valence electrons. Notably, the Fe–S_4_ site of rubredoxin was not assigned any aspherical scattering factors (Figs. 2 ▸*a*–2 ▸*e*), which explains the residual (positive and negative) density in TAAM-based refinements at this site. These features remain to be studied in further detail by quantum-mechanical computations.

## 4. Conclusions

In this paper, we have described how data collection at sub-Ångström resolution has been made as routinely feasible as the collection of standard MX data. Sample preparation, crystallization and post-crystallization treatments remain the most severe roadblocks, but should one overcome these obstacles, the subsequent steps towards ultrahigh-resolution structures displaying deformation densities have been enabled by recent technical innovations comprising high-*Z* detectors, high-energy beamlines delivering dynamically sizable top-hat beams, automated data collection in optimized multiple orientations and a high-quality data-processing pipeline. These technological innovations have been thoroughly tested and validated to the degree that high-quality, sub-Ångström experiments are now possible with the setup described here in a routine manner and are accessible to non-expert users. Of note, dose-aware experimental data-collection strategies such as those described here are beneficial not only for sub-Ångström data collection, but also for use in routine crystallographic experiments, metalloproteins and ligand campaigns (Garman & Weik, 2023 ▸; Ehler *et al.*, 2025 ▸). We would like to emphasize that existing and planned fourth-generation synchrotron sources will offer superior capabilities for such high-energy experiments, *provided coherent optics are incorporated in beamlines in such a manner that adaptable, top-hat beams can be generated and matched on-the-fly to crystal dimensions*.

A significant challenge for the future is the establishment of numerous biological systems that are able to deliver crystals diffracting to sub-Ångström resolution and answer biological questions that can be uniquely addressed with such high-resolution data. Systems that immediately come to mind are the multitude of natural and designer enzymes, where many mechanistic details and quantum phenomena along the reaction trajectory are still elusive. In our hands, we have succeeded in establishing at least 15 systems in our laboratory (unpublished work) that diffract to 0.7 Å resolution or better and the investigation of which massively benefits from the degree of automation and reproducibility described here.

Having acquired such data, efficient procedures to refine high-quality sub-Ångström resolution models are mandatory. In the course of the work described here, we found it essential to rely on the *aB_autorefine* interface, which runs through a defined sequence of steps of *BUSTER* refinements that converge to a final model in a systematic, efficient and reproducible way. Here, we have described the extension of these refinement procedures by using TAAM within *BUSTER*, which enables them to routinely deal with deformation density.

A recent publication (Femoen *et al.*, 2026 ▸) describes the *pyDiSCaMB *package that enables the use of multipolar scattering factors in *Phenix*, including an illustration of calculated deformation density features at 0.7 Å resolution for a tyrosine residue in crambin. Here, we have demonstrated that our experimental and computational methods are capable of supporting the visualization of aspherical features *directly in experimental maps* and that they are well represented by a model refined against 0.43 Å resolution data using TAAM via DiSCaMB in *BUSTER*.

Our experiments further suggest that more detailed electronic structure models of the Fe–S_4_ site of rubredoxin can be tested against the data we report here. In the course of this work, limitations in the contrast of aspherical density features have become acutely apparent. These include not only issues with Fourier series-termination effects, but also the fit of the model to the data. This is most apparent in the inadequacies of solvent models, which have manifested themselves in our hands through poorer fit of the model to data, especially through a large fraction of log-likelihood outliers in the low-resolution range. Solvent models will need major improvements if higher contrast in aspherical features is to be achieved. Should one overcome these obstacles, it is highly likely that extensions of aspherical refinements from TAAM to perhaps even higher order descriptions of electron density, such as the recently reported fragHAR (Bergmann *et al.*, 2026 ▸), or a similar method implemented in DiSCaMB, will be required to derive detailed functional properties. In addition, nuclear densities and their accompanying ADPs through neutron diffraction experiments would be very complementary to the accurate electron-density studies described here. In particular, the dynamic nuclear polarization (DNP) contrast-variation experiments come to mind, which do not require deuteration (Pierce *et al.*, 2020 ▸).

## Supplementary Material

PDB reference: *Pyrococcus abyssi* rubredoxin, 0.43 Â resolution, transferable aspherical atom refinement, 30oh

PDB reference: independent atom model refinement, 30or

## Figures and Tables

**Table:** (*a*) Data collection. Values in parentheses are for the highest resolution shell.

Space group	*P*2_1_2_1_2_1_
*a*, *b*, *c* (Å)	24.730, 39.237, 45.140
α, β, γ (°)	90, 90, 90
Wavelength (Å)	0.38573
Diffraction limits† (Å)	0.441 along a*, 0.462 along b*, 0.456 along c*
Resolution range (Å)	26.62–0.433 (0.464–0.433)
Total No. of reflections	6545565
No. of unique reflections	245905
Ellipsoidal completeness (%)	96.2 (59.9)
Spherical completeness (%)	84.4 (22.3)
Mean *I*/σ(*I*)	23.9 (1.7)
CC_1/2_	1.000 (0.621)
*R*_p.i.m._ (%)	1.1 (45.4)

**Table:** (*b*) Refinement.

	Independent atom model (IAM)	Transferable aspherical atom model (TAAM)
Resolution range (Å)	20.92–0.434	20.92–0.434
PDB code	30or	30oh
*R*_work_/*R*_free_ (%)	6.82/7.25	6.51/6.93
No. of atoms		
Total	1364	1360
Protein (including H atoms)	1191	1187
Solvent	173	173
Average *B* factors (Å^2^)		
Protein (including H atoms)	3.56	3.84
Na atoms	4.66	4.64
Waters	10.13	10.53
Fe atom	1.60	1.60

† Diffraction limits are defined as the inverse length of principal axes of the ellipsoid fitted to the actual anisotropic cutoff surface. The resolution range is defined by the anisotropic cutoff surface. See https://www.staraniso.globalphasing.org and https://www.rcsb.org/news/news/60638da1931d5660393084c3 for further details.

## Data Availability

Structures were deposited in the PDB under accession codes 30or (IAM refinement) and 30oh (TAAM refinement).
